# Developing a synthetic national population to investigate the impact of different cardiovascular disease risk management strategies: A derivation and validation study

**DOI:** 10.1371/journal.pone.0173170

**Published:** 2017-04-06

**Authors:** Josh Knight, Susan Wells, Roger Marshall, Daniel Exeter, Rod Jackson

**Affiliations:** 1 Centre for Health Policy, School of Population and Global Health, University of Melbourne, Melbourne, Australia; 2 School of Population Health, Faculty of Medical and Health Sciences, University of Auckland, Auckland, New Zealand; Hunter College, UNITED STATES

## Abstract

**Background:**

Many national cardiovascular disease (CVD) risk factor management guidelines now recommend that drug treatment decisions should be informed primarily by patients’ multi-variable predicted risk of CVD, rather than on the basis of single risk factor thresholds. To investigate the potential impact of treatment guidelines based on CVD risk thresholds at a national level requires individual level data representing the multi-variable CVD risk factor profiles for a country’s total adult population. As these data are seldom, if ever, available, we aimed to create a synthetic population, representing the joint CVD risk factor distributions of the adult New Zealand population.

**Methods and results:**

A synthetic population of 2,451,278 individuals, representing the actual age, gender, ethnicity and social deprivation composition of people aged 30–84 years who completed the 2013 New Zealand census was generated using Monte Carlo sampling. Each ‘synthetic’ person was then probabilistically assigned values of the remaining cardiovascular disease (CVD) risk factors required for predicting their CVD risk, based on data from the national census national hospitalisation and drug dispensing databases and a large regional cohort study, using Monte Carlo sampling and multiple imputation. Where possible, the synthetic population CVD risk distributions for each non-demographic risk factor were validated against independent New Zealand data sources.

**Conclusions:**

We were able to develop a synthetic national population with realistic multi-variable CVD risk characteristics. The construction of this population is the first step in the development of a micro-simulation model intended to investigate the likely impact of a range of national CVD risk management strategies that will inform CVD risk management guideline updates in New Zealand and elsewhere.

## Introduction

While not currently widely established in the health field, the use of microsimulation is growing [1–4] and the technique has been in use in other areas of analysis and research for a number of decades[5]. Common in fields such as economics[6], policy analysis [7] and engineering [8]; simulation is often used where the ability to experimentally test an idea is not feasible due to cost, time constraints or ethical/legal considerations. A key distinction of microsimulation is that within the model each ‘elemental decision making unit’, as Orcutt put it [5], is indivisible; typically in health this unit is a person. This contrasts with other forms of simulation where cohorts of individuals; with a shared set of characteristics (determined by the goal of the simulation), typically representing average values of the population of interest, are simulated. As microsimulations are on an individual basis each synthetic ‘person’ has a set of characteristics assigned to them individually which allows for the greater capture of the heterogeneity of population characteristics, something not possible with a cohort, which is important in the assessment of many healthcare interventions.

The requirement for individual level data however can be a barrier to undertaking a microsimulation analysis. The scope and quality of individual level data available can be limited, particularly where the simulation is on a national or regional scale, as large amounts of detailed information on a broad cross-section of the population is required. When the microsimulation involves a health application the likelihood of privacy issues arising is high; creating issues with access to key data and further problems with the wider dissemination of this data among analysts and researchers for collaboration and replication of results. To overcome the hurdles of the high data requirements and the privacy implications of individual level data a synthetic population, consisting of generated data that closely resembles the real population, can be developed to be used within the microsimulation.

Where all the required characteristics are represented in a single, aggregated source a synthetic population can be developed from a single data repository data [9, 10], often census data. When there is no single source which has data on all the required characteristics a synthetic population can be developed from a number of data sources [11, 12]. The advantage of combining information from multiple sources is that each individual repository can contain high quality data about one or more of the variables required in the population to be simulated without a requirement for it to be directly linked to all other repositories. This method of combining data, when undertaken carefully, can allow for a more complete dataset to be developed than is available in any single existing data source and is particularly appropriate for population studies of cardiovascular disease (CVD) as the condition has a complicated aetiology stemming from multiple risk factors [13]. To maintain the appropriate correlations between datasets there must be variables in common between the datasets. This allows for the appropriate combining of the separate information sources. The correlation between the datasets will only be maintained on the basis of the variables in common between datasets (e.g. age, gender, ethnicity and disease status) so maximising the ‘overlap’ of variables between datasets is beneficial.

For the preventive management of CVD there is now broad consensus among researchers and policy makers that the optimal clinical strategy should be based primarily on a person’s multi-variable predicted absolute CVD risk rather than on the basis of individual risk factor levels [13–15]. There is however less agreement on the most appropriate method of predicting CVD risk or the thresholds at which treatment should be initiated. Our study was inspired by a UK project that used microsimulation to compare two CVD risk management strategies; a multi-variable risk-based strategy and one based simply on treating all people above specified age thresholds. The number of people in the United Kingdom (UK) who would meet treatment criteria and the number of CVD events likely to be prevented through treatment was estimated for the two strategies [16]. The investigators developed a multi-variable synthetic population based on UK health survey data and used micro-simulation methods to estimate treatment effects. In contrast to the general trend of national policies, which focus on absolute risk [13, 17–19], the authors concluded that an age-alone strategy for determining treatment assignment was similar in terms of predictive accuracy and was superior on a cost-effectiveness basis compared to a multi-variable strategy. We planned to replicate and extend the UK study in a New Zealand setting, to inform the development of new national CVD risk management guidelines [20, 21].

There were two primary reasons for the development of a new population rather than using the work from the existing study. Firstly the maintenance of the relationships between variables is key in multivariate equations, if there are is a clustering of risk factors the estimates from a populations where the variables are developed via independent draws even from appropriate distributions will not give an accurate result. Secondly, to develop locally relevant outcomes a population reflecting New Zealand rather than English demographics was required.

As the characteristics of the study population are so critical to the outcome of a micro-simulation, the development and validation of these population data was considered a critical step in the process. The goal of this paper is to describe the development and validation of a synthetic New Zealand population.

## Methods

We constructed a synthetic New Zealand (NZ) population aged 30 to 84 years with each ‘synthetic’ person having the complete set of CVD risk factors used in two CVD risk prediction equations; the New Zealand Framingham CVD risk equation [18] and a risk equation recently developed by our research group. This was undertaken in three broad steps, first a demographic framework was created from census data to ensure the representative nature of key demographics. Secondly, disease and medication histories were developed for each individual from linked hospital admission and pharmacy dispensing data. Finally a large cohort of individual level CVD risk assessment data was used to develop required biological variables for all individuals created in the prior steps. Methods allowing the retention of correlations between variables while retaining variable heterogeneity were used to replicate the true population as accurately as possible.

The variables required for the CVD risk equations are: age, gender, ethnicity, social deprivation, smoking status, diabetes status, personal history of CVD, blood pressure and lipid lowering drug treatment, systolic blood pressure (SBP), the total cholesterol to high density lipoprotein cholesterol ratio (TC: HDL), and family history of premature CVD. The overall goal was to create a population of synthetic individuals representing all 2,451,278 people aged 30 to 84 years who completed the 2013 New Zealand census.

Within the methods section the five data sources used in the synthetic population development are first described. The stages of the synthetic population development are then outlined. Finally the steps used to validate the population are described.

### Data sources

Five data sources were used to assign risk factors to the synthetic population,

i The usually resident population count from the 2013 national census [22] cross- tabulated by age, sex, ethnicity, social deprivation and smoking was obtained from Statistics New Zealand. The census data were provided with the age at time of census variable (the only continuous variable) aggregated into single year brackets (see appendix 2 for an example). Individual level census data (unit file data) is available for the New Zealand population however low level aggregated data was preferred as a key goal was to develop a freely distributable synthetic population. The use of individual level census data would have likely entailed additional restrictions on the basis of privacy considerations. As the only continuous variable represented in the census data was age the aggregated nature of the data was not considered to diminish the quality of the remaining variables. The ethnicity categories were; Maori, Pacific Island, Indian, Chinese, Other Asian, Other Ethnicities and European. For those individuals who self-identified with more than one ethnicity in the 2013 census, a single, prioritized ethnicity was generated with the prioritization based on the order listed above. Socioeconomic status was derived from the New Zealand Deprivation Index Score (NZDep), which is a measure assigned to a person’s area of residence. NZDep is based on nine variables from the census, reflecting eight dimensions of relative deprivation of census tracts [23]. For these analyses individuals were assigned to one of five quintiles (from least deprived = 1, to most deprived = 5) of the nationwide distribution of NZDep score. Smoking data was categorised as ‘non-smoker’, ‘ex-smoker’ and ‘current smoker’, coded as 0, 1 and 2 respectively. Due to the Statistics New Zealand confidentialityrules [22] associated with the census data, cells containing small counts (less than 6 individuals) were suppressed (coded ‘..C’). To allow for the inclusion of these individuals a realistic values, based on the censoring rules and existing data, was developed (see appendix 2 for further details). While census data was complete for the age and gender variables, some individuals were assigned a ‘not otherwise known’ ethnicity or NZDep status. As everyone in the synthetic population required complete data, missing variables were assigned based on the proportion of known ethnicities and NZDep in the census population.
ii The National Minimum dataset (NMDS) contained individual patient hospitalisation data on all NZ public hospital discharges coded according to The International Classification of Diseases, version 10 Australian Modification (ICD-10 AM). This dataset was used to derive a patient history of prior CVD (appendix 1 lists the ICD codes used). Comprehensive national hospitalisation data was available starting from 1993 through to the end of 2013, with significant partial data available from 1986.
iii The national Virtual Diabetes Register (VDR) [24] is an annually updated database that identifies all New Zealanders believed to have been diagnosed with diabetes, based on an algorithm that combines national laboratory test reimbursement data (indicating HbA1c testing) with diabetes-related dispensing and hospital admission data.
iv The national pharmaceutical dispensing database (PHARMS) records all subsidised and controlled drugs dispensed by community pharmacies in NZ. All commonly used CVD preventive medications in NZ are subsidised so we were able to derive medication data for the two main classes of CVD preventive medication (i.e. lipid lowering and blood pressure lowering medications) from this database. Reliable recording of individual patient dispensing episodes has increased over the last decade from 64% in 2004, to 92% in 2006 to over 96% from 2010 onwards and currently captures virtually all dispensing episodes (S. Ross, Ministry of Health, personal communication, 2014).
v The PREDICT primary care CVD risk factor dataset [25] was used to derive the remaining CVD risk factors required for the risk prediction models, namely: SBP, TC:HDL ratio and family history of premature CVD. This dataset is generated from the PREDICT web-based CVD risk assessment and management decision support system used by approximately 80% of general practitioners in the Auckland and Northland regions of New Zealand (approximately 35–40% of all New Zealand general practitioners). A 2013 PREDICT dataset extract contained the baseline CVD assessments of 272,645 people with complete data recorded on age, sex, ethnicity, NZDep, smoking status, diabetes, SBP, TC:HDL ratio, prior personal history of CVD and family history of premature CVD. The dataset included approximately 50% of all people eligible for risk assessment in the region, but was biased towards higher risk population groups (such as older age groups, people with diabetes and Maori, Pacific and Indian ethnic groups)[25], so could not be used alone to derive the synthetic population.

The four non-census data sources listed above (ii-v) all include individually identifiable person level data that are able to be linked using an encrypted version of the National Health Index (NHI) number. The NHI is a unique national identifier number is attached to records of publicly funded and subsidised health service interactions for over 98% of New Zealanders as well as a range of demographic data (e.g. date of birth, gender, ethnicity and NZDEP). The encrypted nature of the NHI meant that the individual level data used in this study would not be able to be further linked to any information coded with an unencrypted NHI; helping, along with other safe guards, to maintain the anonymity of the individuals in the dataset. New Zealand ethics committees allow secondary re-use of health data without individual patient consent where data are not identifiable. Information about the PREDICT study is available at all general practice locations, and patients may opt out of having their de-identified data being included in the cohort. The PREDICT study was approved by the Northern Region Ethics Committee Y in 2003 (AKY/03/12/314) with subsequent annual approval by the National Multi-region Ethics Committee since 2007 (MEC07/19/EXP).

### Synthetic population generation

In summary; the first step was to construct a synthetic population in which the joint distributions of the demographic variables *age*, *sex*, *ethnicity* and *SES* were exactly matched to the distribution of these factors in the 2013 NZ Census (data source [i] above). This first step was largely deterministic, with age being the limited exception, based on the fact that there was low measurement uncertainty or variability over time for these ‘demographic’ characteristics.

The second step involved using Monte Carlo sampling and multiple imputation to randomly assign the remaining variables: *smoking*, *diabetes*, *prior CVD*, *medications*, *SBP*, *TC*:*HDL ratio*, *and family history*, *conditional on the Census demographic variables*. These ‘non-demographic variables’ were assigned at using a Monte Carlo method based on observed proportions to capture the inherent variability of these characteristics.

#### Demographic variables

The initial stage of the development involved setting up a realistic structure for the New Zealand population. This was done by moving through the aggregated census data supplied by Statistics New Zealand (see example in appendix 2) row-by-row with the goal of creating a representative synthetic population were each individual was represented by a row in the dataset. Each strata or row was defined by an age, sex, ethnicity and SES group. The number of individuals in the strata was found by summing the smoking statuses to get a total number of individuals represented.

Age was assigned by random draw of X values from a uniform distribution bounded by Y and Y+1 where Y is the minimum age group and X is the number of individuals in the age/gender/ethnicity group. Sex, ethnicity and SES were then deterministicallyapplied per strata as there was no variation.

#### Non-demographic variables

The non-demographic variable distributions in the synthetic were developed as follows:

i Smoking was assigned in a Monte Carlo fashion to one of the three potential outcomes (‘current smoker’, ‘ex-smoker’ and ‘non-smoker’) according to the observed frequency of smoking categories in the 2013 census, conditional on age, sex, ethnicity and NZDep. Despite this data coming from the census a Monte Carlo method was used in preference to creating further strata based on smoking and deterministically assigning smoking status. This was on the basis that self-report of smoking is less reliable than the other variables defined by the census data and that smoking was thought to have greater variability over time (i.e. it is more likely your smoking status will change that your age, gender, ethnicity and SES).
ii Diabetes and prior CVD statuses were jointly derived due to their strong association. We first linked previous CVD hospitalisations (from data source [ii] above) and the VDR (data source [iii]) via the encrypted NHI using a database which also included age, gender and ethnicity which gave a joint probability of disease state. A disease status was then probabilistically assigned to each individual (CVD only, diabetes only, diabetes and CVD, or neither) using Monte Carlo sampling, conditional on age, gender and ethnicity.
iii Medication status for lipid lowering (LL) and blood pressure lowering (BPL) drugs was derived using a similar approach to the diabetes and prior CVD variables. The proportions of people taking these medications came from the national drug dispensing database (data source [iv]). A current medication status (LL and BPL drugs, LL drugs only, BPL drugs only, or neither) was assigned to each individual using Monte Carlo sampling, conditional on age, sex, ethnicity and history of CVD. Further details of steps i-iii are provided in appendix 2.

#### Imputed variables

iv The final step was to assign a SBP, TC:HDL ratio, and a family history of premature CVD derived from the individual-level PREDICT data (data source [v]) utilising multiple imputation [26–28]. This was done by treating the remaining variables as ‘missing’ for the purposes of the imputation process. Conventionally imputation has been used to replace missing data with plausible values given the known or non-missing data. In this circumstance the PREDICT data was used as the ‘known’ data whereas all required data values (i.e age, gender, ethnicity as well as SBP, TC:HDL ratio, and a family history of premature CVD) were established. The synthetic data contained the variables previously developed in steps i-iii but ‘missing’ values for SBP, TC:HDL ratio, and a family history of premature CVD. To undertake the imputation the PREDICT dataset was appended to the synthetic population to create a single dataset containing both real (PREDICT) and synthetic data allowing the generation of realistic values for the synthetic population based on real world data. Further details of the imputation process are provided in appendix 3 and a summary of the use of the datasets is suppled in Table 1.

**Table 1:** Summary of data sources and contribution to synthetic population.

Data Source Name	Source	Variables derived from data source	Linked by
New Zealand Census	Statistics New Zealand	Age, Gender, Ethnicity, SES, Smoking	Framework created from this dataset
National Minimum dataset	New Zealand Ministry of Health	History of CVD status	Age, ethnicity, gender
National Virtual Diabetes Register	New Zealand Ministry of Health	Diabetes status	Age, ethnicity, gender
National pharmaceutical dispensing database	New Zealand Ministry of Health	Blood pressure and lipid lowering medication	Age, ethnicity, gender, history of CVD, diabetes status
PREDICT primary care CVD risk factor dataset	VIEW/PREDICT CVD risk screening project	SBP, TC:HDL ratio family history of CVD	Age, ethnicity, gender, history of CVD, diabetes status, Blood pressure and lipid lowering medication status

### Validation

We then followed a two-stage validation process; first validating against the datasets used to develop the synthetic population (internal validation) and then against datasets which had not been involved in the development of the synthetic population (external validation). The internal validation simply compared the synthetic population variable distributions to the equivalent distributions in datasets used to generate the synthetic population, to ensure that the synthetic data reflected the source data and the relationships between the variables. The synthetic population was then externally validated by comparing the risk factor distributions generated in the synthetic population with equivalent data from external sources not used in the development of the synthetic population. Internal validation, but not external validation, was done for the age, sex and ethnicity variables as these data were derived directly from the NZ Census, which was considered to be the gold standard. Only internal validation was possible for current drug treatment and family history of CVD, as no relevant external datasets were available for these variables.

The data used for external validation was sourced from the published studies conducted in the New Zealand population. As these data were only available in an aggregated form, this placed some restrictions on how it could be compared with the synthetic population data. In cases where high quality, recent national level data was not available, regional studies were used. To enable valid comparisons, the age and ethnicity aggregations in the synthetic population had to be modified to match those in the external validation data. Mean values from the external validation sources were then plotted against equivalent values from the synthetic population. Where possible, 95% confidence intervals for the external validation data were also plotted.

In situations where the synthetic and external data used for the validation were clearly different, a second external data source was sought. If the two external sources were consistent but were different from the synthetic data distributions, then the synthetic data was modified *post-hoc* so that the mean values of the synthetic and external data were similar, see appendix four for further details.

The analysis and all other the computational work was undertaken in the R 3.1.3 computing environment [29] and used the MICE [26] and dplyr [30] packages extensively.

## Results

Table 2 presents a summary of the synthetic population variables by age group. Europeans accounted for the majority of the population in all age groups and this proportion increasing with age, while the proportions of all other ethnic groups decreased with increasing age. Smoking prevalence and familial history of premature CVD decreased with increasing age whereas diabetes prevalence, prior CVD, mean SBP, and medication usage increased with advancing age.

**Table 2:** The synthetic population variable distributions by age group.

Variables		Age				
		30–44	45–54	55–64	65–74	75–84
Number of individuals		828,518	600,532	491,463	344,559	186,206
Male (%)		47.3	48.0	48.7	48.4	45.3
Ethnicity(% of age)	Maori	13.9	12.0	9.5	6.8	4.5
	Pacific	6.1	4.8	3.6	2.7	1.9
	Indian	5.0	3.1	2.4	1.6	0.9
	Chinese	4.6	3.6	3.6	2.6	2.2
	Other Asian	4.9	3.5	2.0	0.8	0.4
	Other	1.6	0.8	0.5	0.2	0.1
	European	63.9	72.3	78.5	85.2	90.0
Deprivation Quintile(1 is least deprived)	1	20.9	25.1	24.5	22.5	19.2
	2	21.2	22.0	21.9	21.6	20.5
	3	20.7	19.6	19.9	20.4	20.9
	4	19.5	17.4	18.1	19.3	22.2
	5	17.6	15.9	15.7	16.2	17.2
Smoking	Current	18.9	17.7	13.8	9.9	6.6
	Ex-smoker	22.3	25.3	30.1	35.1	35.0
	Never smoked	58.9	57.0	56.0	55.0	58.5
Diabetes (%)		4.1	8.2	13.8	19.4	22.6
Prior CVD (%)		0.6	2.5	6.1	12.6	22.4
LL medication (%)		1.6	8.2	20.5	34.8	41.3
BPL medication (%)		3.1	12.7	28.5	47.4	60.6
SBP(mean, sd)		122.7 (17.3)	127.9(17.2)	132.5(17.3)	136.9(17.4)	140.4(17.5)
TC:HDL(mean, sd)		4.0(1.4)	4.2(1.2)	4.1(1.2)	4.0(1.1)	3.8(1.1)
Family history of premature CVD (%)		16.5	13.5	12.1	10.6	8.5

### Age, gender and ethnicity (Internal Validation Only)

**Fig 1:**
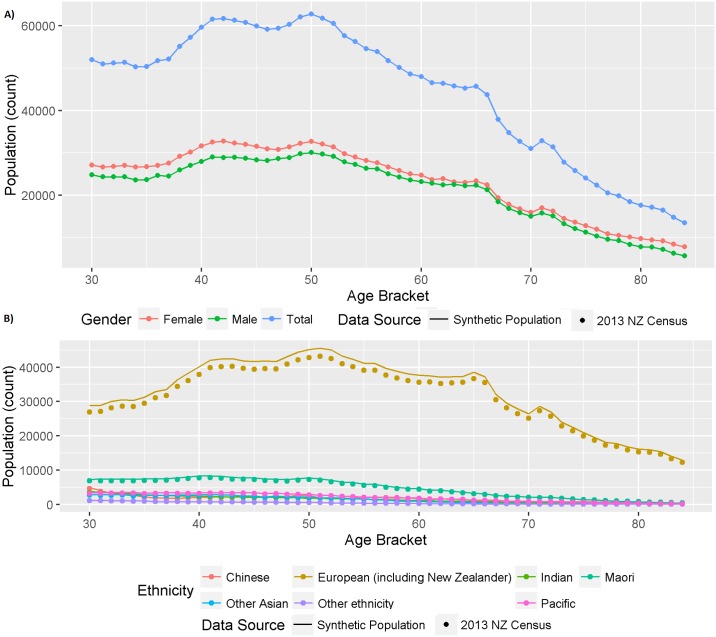
Internal validation comparing the synthetic (line) and 2013 Census population (dots) by one-year age group counts (A) for the total population and gender, and (B) for each ethnic group.

Fig 1a demonstrates that the synthetic population (illustrated the solid lines) is, as expected, identical to the 2013 Census population (the dots) by age and gender.

Fig 1b shows that the ethnicity by age group patterns of the synthetic data (dots) are also mirrored in the Census population (solid lines), although the synthetic population numbers are slightly higher. The lack of an exact match is due to the assignment of individuals in the Census population with missing ethnicity data to existing ethnicity categories (described in the Methods).

### Smoking prevalence by age, gender and ethnicity (Internal & External Validation)

**Fig 2:**
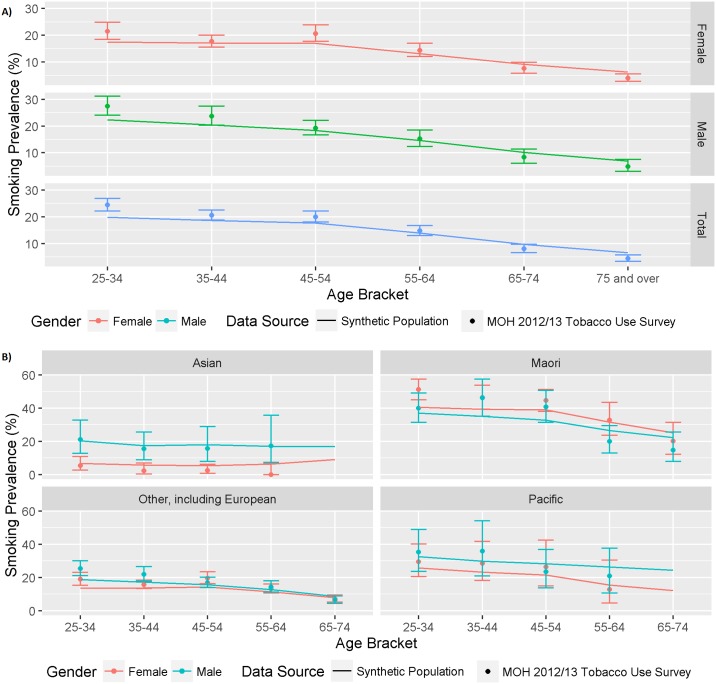
External validation comparing smoking prevalence by gender and age in the synthetic population (line) and the 2012/13 MoH Tobacco survey (points and 95% CIs) (A) the total population and (B) separate ethnic groups.

The synthetic population smoking prevalence distributions by age, gender, ethnicity and deprivation showed close correlation with the Census population smoking data used to generate it (see Figure A-C in S5 File). There was also good agreement on smoking rates between the synthetic population and the NZ Ministry of Health (MoH) 2012/13 Tobacco Use Survey [31], by age, gender and ethnicity (Fig 2a and 2b). Fig 2a illustrates a declining trend in smoking prevalence with increasing age in both men and women in the synthetic population (line) and MoH data (points and 95% CIs). While there was a very good level of agreement throughout the middle age bands, there was a slightly lower estimate in the youngest age band and higher estimate in the oldest age band in the synthetic population compared to the MoH survey population. This is likely to be due to the exclusion of people aged 25–30 years and over 85 years from synthetic population but not from the MoH data.

Fig 2b compares smoking prevalence by age, gender and ethnicity in the MoH survey and synthetic population and also shows reasonable agreement. Due to the restricted sample size in the MoH survey, some ethnic/gender/age groupings have wide confidence intervals. Previously reported ethnic differences in smoking prevalence were observed, including the high smoking prevalence in Maori, particularly females, and the very low prevalence in Asian females.

### Diabetes prevalence by age, gender and ethnicity (Internal & External Validation

**Fig 3:**
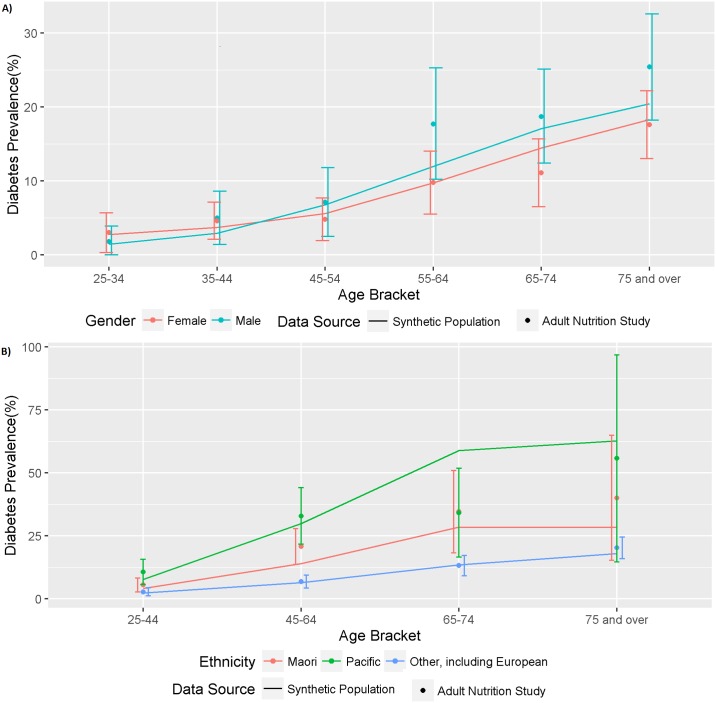
External validation comparing diabetes prevalence in the synthetic population (line) and the 2008/9 National Adult Human Nutrition Survey (points and 95% CIs) by age group. (A) the total population and (B) separate ethnic groups.

There was very close agreement between the synthetic population diabetes prevalence and the national Virtual Diabetes Register (VDR)-based prevalence estimates used to generate it (see Figure D in S5 File). Similarly, in the external validation, there was reasonably good agreement between diabetes prevalence in the synthetic population and in the New Zealand 2008/09 Adult Nutrition Survey [32] by age and gender, for the total population (Fig 3a) and by ethnicity (Fig 3b). The ethnicity data from the New Zealand 2008/09 Adult Nutrition Survey was categorised as Maori, Pacific and New Zealand European/Other (NZEO). While there were some small differences, all synthetic population values fell within the 95% confidence intervals of the survey-based prevalences, with the exception of Pacific individuals between the ages of 65 and 74. The overall fit of the synthetic data, compared to the nutrition survey, was adequate and the reliability of the VDR data (being a nationwide, regularly updated database) was preferred to the survey data so no post-hoc modification was made to the prevalence of diabetes in the synthetic population.

### Personal history of CVD by age, gender and ethnicity (Internal Validation)

**Fig 4:**
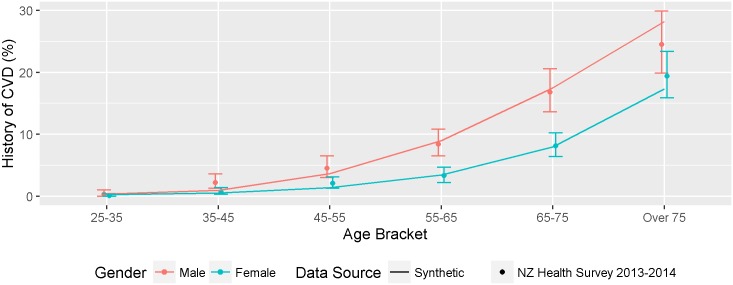
External validation comparing the prevalence of previous CVD by gender and age in the synthetic population (line) and data from the 2013/14 Zealand health survey (points and 95% CIs).

The synthetic population accurately reflected the prevalence of a personal history of prior CVD derived from the national hospitalisation data stratified by age, gender and ethnicity (see Figure E in S5 File). In the external validation, the synthetic population was compared to a national CVD prevalence study conducted by the New Zealand Ministry of Health during the 2013–14 New Zealand health survey [33]. As shown in Fig 4, the prevalence of CVD in synthetic population was similar to the prevalence observed in the health survey by age and gender.

The Health Survey reported age-adjusted prevalence ratios by ethnicity, rather than prevalence proportions, which we compared with age and gender adjusted odds ratios for Maori, Pacific Island and Asian populations derived for the synthetic population using logistic regression controlling for age, gender and the binary ethnicity variable.

For people who identified as Maori in the synthetic population, the OR for prior CVD was 1.58 compared to non-Maori, which was similar to the Health Survey adjusted prevalence ratio of 1.57 (95% CI 0.1, 25.71). For Pacific people in the synthetic population, the odds ratio was 1.85, compared to a prevalence ratio of 1.71 (95% CI 0.1, 28.55) in the Health Survey, and for Chinese and Other Asians combined, the adjusted OR was 0.43 in the synthetic population and 0.58 (95% CI 0.03,9.92) in the Health Survey.

### Systolic Blood Pressure (SBP) by age, gender and ethnicity (Internal and External Validation)

**Fig 5:**
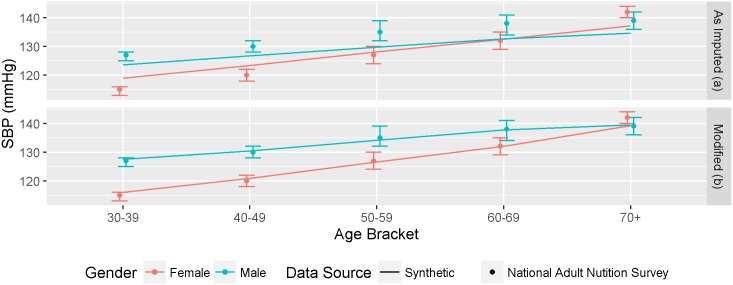
External validation comparing systolic blood pressure levels by age and gender in the synthetic population (lines) and the 2008/9 National Adult Nutrition Survey (points and 95% CIs) (A) as imputed and (B) modified.

The SBP variable in the synthetic population was imputed using data from the PREDICT dataset[25]. The internal validation of systolic blood pressure levels by age and gender (Figure F in S5 File) indicated a close alignment between the synthetic population and the PREDICT dataset except for women under about 45 years of age (note: there were very few younger women in the PREDICT dataset). However in the external validation against independent national [34] and regional surveys [35], the gender differences in SBP in the synthetic population were not as pronounced as generally observed in other studies (Fig 5a).

Therefore, a post-imputation adjustment (Fig 5b) was made to the SBP to bring the synthetic population data into alignment with previously reported data. The adjustment shifted the age and gender specific mean levels in the population while retaining the variability developed during the imputation process (adjustments described in Appendix 4). Following these adjustments, the synthetic population SBP distributions were very similar to those for the National Survey data (6b).

### TC:HDL ratio by age and gender (Internal and External Validation)

**Fig 6:**
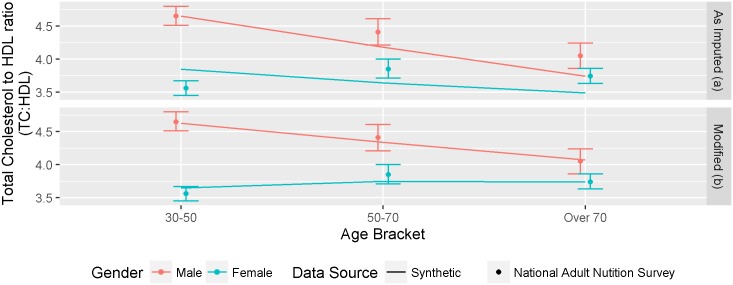
External validation comparing TC:HDL ratio levels by age and gender in the synthetic population (lines) and the 2008/9 National Adult Nutrition Survey (points and 95% CIs) (A) as imputed and (B) modified.

The TC:HDL ratio variable in the synthetic population was imputed using data from the PREDICT dataset [25]. As with SBP, the synthetic population distributions showed good agreement with the PREDICT population distributions by age and gender, except in younger women (see Figure G in S5 File). However the TC:HDL ratio distributions in the synthetic population showed less agreement with an external data source of 2.9 million TC:HDL ratio tests from a regional laboratory database, measured between 2006 and 2012 and with a second data source, the New Zealand 2008/9 National Adult Human Nutrition Survey findings (Fig 6a). As there was reasonable agreement between the two external data sources, the synthetic population TC:HDL ratio distributions were adjusted to match the distribution observed in the laboratory data (adjustments described in Appendix 4). The primary modification was a reduction in the ratio of younger individuals who are not as well represented in the PREDICT dataset.

Following the modification, the distribution more closely resembled the data from the 2008/9 National Adult Human Nutrition Survey (Fig 6b).

### Medication and familial history of premature CVD

We found close agreement between the synthetic and source datasets for LL and BPL medication status (see Figure H in S5 File) but there was no relevant data available for an external validation. The medication data was derived from a comprehensive, high quality national database, which like the census demographic data is essentially the gold standard and as such the lack of external validation was considered acceptable.

The internal validation of the familial history of premature CVD (derived from the PREDICT database) showed close agreement with the synthetic population distributions by age, gender and ethnicity (see Figure I in S5 File). However, no external validation was possible due to a lack of a relevant external data source. As the PREDICT data [25] is by far the largest New Zealand dataset that measured this variable, it is considered to be the best current source of this data.

## Discussion

This study has demonstrated that it is possible to develop a synthetic population with demographic and CVD-risk profiles similar to the New Zealand adult population, using national census data, routine national hospitalisation and medication data and a large sub-national primary care dataset. The aim of this study was to produce a synthetic population that could be used to run simulations to investigate the likely impact of a range of national CVD medication treatment policy options, on the numbers of New Zealanders meeting treatment criteria and on the likely number of CVD events that would be prevented. If these simulations are to produce realistic and reliable outputs, it is essential they are applied to population data that accurately represent the multi-variable CVD risk status of New Zealanders. The ‘individual’ level data generated were not intended to match any particular real world person, however as indicated by the validation analyses performed, the synthetic population generally matches the socio-demographic and CVD risk characteristics of the New Zealand adult population.

A strength of this study is the use of a methodology that combines total population data from a national census and national routine hospitalisation and drug dispensing databases, together with micro-level data from a large scale sub-national primary care database. The primary care data used in this study were collected recently in two regions that contain approximately one-third of the New Zealand population, thus increasing the likelihood of developing a valid representation of the New Zealand population. The external validation of the synthetic population in regard to key CVD predictors is also a strength of this study and gives us confidence that the synthetic population can be used for assessing the impact of different CVD risk management treatment options. Currently there is no equivalent single comprehensive survey-based dataset in New Zealand and to the authors’ knowledge this synthetic population is the most complete and comprehensive multi-variable CVD risk dataset representing a national population available.

The synthetic population is only as good as the quality of the data used to generate it and the two continuous variables–systolic blood pressure and the TC:HDL ratio–required adjustment to more accurately reflect the mean values reported in the external data sources used in the validation process. While the internal validation confirmed that both these variables were imputed correctly, the age and gender distributions in the PREDICT dataset used to generate them differed somewhat from published studies. The difference was particularly pronounced in younger women who had higher SBP levels and higher TC:HDL ratios than observed in the external data sources. This was not totally unexpected as there were very few younger women (under the age of 45) in the PREDICT dataset because they do not meet nationally recommended criteria for CVD risk assessment unless they are known to be at high risk. The presence of the records of the woman of a younger age in the PREDICT dataset is a potential indication that they are a-typical in terms of CVD risk hence the screening at a earlier than recommended age. There also appeared to be some measurement error in the SBP levels recorded in the PREDICT dataset used to impute the synthetic population SBP distributions. The PREDICT blood pressure measurements were mainly taken by busy general practitioners and practice nurses who tend to show digit preference by rounding measurements to the nearest 10 mmHg[36]. It is likely that this rounding error reduced the magnitude of the differences in blood pressure between men and women usually observed in research studies where blood pressure is measured and recorded more accurately. The modifications to variables were required made were kept as simple as possible and undertaken in a manner which retained the variability provided by the imputation process. A sensitivity analysis will be undertaken in the simulation phase using both the adjusted and unadjusted data.

We were unable to externally validate the medication or family history of premature CVD variables as there were no relevant national or regional survey data available. The medication data were derived from the national PHARMS database that documents all subsidised drugs dispensed from community pharmacies in New Zealand. As all commonly used CVD preventive medications are subsidised in New Zealand and reimbursement for pharmacists is via the reported data the level of completeness is thought to be very high. The quality of the family history of CVD variable is less certain, however this variable is a relatively weak predictor in the CVD risk prediction equations that will be used in treatment simulations and any inaccuracies will have minimal influence on the outputs.

The census data used in developing the synthetic population was based on the ‘usually resident population’ count. Statistics New Zealand also develops an ‘estimated resident population’ which manages issues such as under counting due to non-response or to missing data on particular census questions, such as ethnicity. However, the estimated resident population data available was not suitable for this study as known high risk ethnic populations (Pacific and Indian individuals) were aggregated with lower risk populations. Within the census section of the synthetic population development, individuals with missing ethnicity data were assigned an ethnicity based on the reported ethnicities proportions by age and gender strata. Although this approach is less sophisticated than the techniques used by Statistics New Zealand in developing the estimated resident population, it does partially address the ethnicity non-response issue while providing ethnicity data in the correct format.

This population contains all the key variables required to calculate the estimated CVD risk for individuals based on the most common equations in use in New Zealand. There are a number of variables which could be considered ‘unobserved’ which might modify CVD risk or medication efficacy. While their addition would be useful, and is planned for the future, their absence should not impact on the intended simulation. Within the micro-simulation an equation with pre-defined variables will be used to estimate risk which will have incorporated the impact of the ‘unobserved’ variables so this is not thought to be a detriment to the current work. As more integrated health data becomes available likely that it will be possible to develop further variables for both CVD and other diseases.

As a function of the development process there will be minor variations between the synthetic populations. Characterisation of this intra-population variation allows for an estimation of the uncertainty of the synthetic population development. For the more numerous sub-populations, for example individuals of European ethnicity, the intra-population variability of summary values would be expected to be small. For smaller sub-populations, for example the older age brackets of Maori and Pacific individuals, a greater degree of variability between populations would be expected due to Monte Carlo error. This should be taken into account, and the variability quantified, if summary data is being calculated from the synthetic populations. To this end multiple synthetic populations will be freely provided to allow and assessment of the intra-population variability of any subsets of interest.

Although this population is synthetic, the external validation process indicates that it provides a reasonable proxy for the multi-variable CVD risk factor levels of adult New Zealanders. Moreover, it is possibly the most current and comprehensive nationally representative individual ‘person’ dataset of multiple CVD risk factors available. Due to its synthetic nature the population is free of privacy issues and as such is able to be shared much more freely than actual patient data. While the initial goal is to use this synthetic population to facilitate CVD simulations for assessing the impact of different treatment strategies, there has already been interest from research groups to use it for other applications. Future work is likely to involve extending the population both to include new risk factors for both CVD and other diseases of such as cancer, and we plan to make these data available to other researchers and health service planners.

## Supporting information

S1 File ICD-10 codes used to define a history of CVD. (DOCX)

S2 File Monte Carlo process description. Further information on the Monte Carlo process used during the population development.
(DOCX)

S3 File Imputation process description. Further information on the imputation process used during the population development.
(DOCX)

S4 File Blood pressure and TC:HDL ratio adjustment description. Description of the adjustments undertake to modify synthetic blood pressure and lipid data.
(DOCX)

S5 File Additional internal validation plots excluded from the main text. Nine additional plots of internal validation exercises undertaken.
(DOCX)
