# Supplementary material for: Developing a synthetic national population to investigate the impact of different cardiovascular disease risk management strategies: A derivation and validation study
Source: PLoS One. 2017 Apr 6;12(4):e0173170. doi: 10.1371/journal.pone.0173170 (PMC5383032; doi:10.1371/journal.pone.0173170)
Supplement: S1 File — (DOCX) [file pone.0173170.s001.docx]

## Supporting Information 1.

## ICD-10 codes used to identify CVD events in medical records

ICD-10 hospitalisation codes used to define prior CVD. The likelihood of an individual having a history of CVD is defined by events which resulted in a hospitalisation and that one of the ICD codes associated with the event is listed in this list. Events that did not eventuate in a hospital admission or were not codes with an appropriate code will not be recorded as an event. The data used spanned 1993 to 2013 and the proportion was developed using individuals who were alive as of 2013 (as defined by not having a date of death or a date of death after 2013 recorded in the national data). This was designed to match the census population on which the synthetic population was developed. If an individual had an event prior to 1993 and has not had one subsequently then this event will possibly not be recorded (although there is partial data extending back to 1986) however that is 20 year of comprehensive follow-up so is thought to capture the vast majority of events.

S1 Table. ICD 10 codes used to define prior CVD

| CLINICALCODE | DESCRIPTIONSHORT |
| --- | --- |
| I210 | Acute transmural MI of anterior wall |
| I211 | Acute transmural MI of inferior wall |
| I212 | Acute transmural MI of other sites |
| I213 | Acute transmural MI of unspecified site |
| I214 | Acute subendocardial MI |
| I219 | Acute myocardial infarction unspecified |
| I220 | Subsequent MI of anterior wall |
| I221 | Subsequent MI of inferior wall |
| I228 | Subsequent MI of other sites |
| I229 | Subsequent MI of unspecified site |
| I230 | Haemopericardium current comp foll ac MI |
| I231 | ASD as current comp following acute MI |
| I232 | VSD as current comp following acute MI |
| I233 | Rupt card wall wo hemopericrd foll ac MI |
| I234 | Rupt chordae tendineae comp foll ac MI |
| I235 | Rupt papillary muscle comp foll ac MI |
| I236 | Atrl thromb auric append ventric w ac MI |
| I238 | Other current complication foll acute MI |
| I240 | Coronary thrombosis not resulting in MI |
| I248 | Other forms of acute IHD |
| I249 | Acute ischaemic heart disease NOS |
| I252 | Old myocardial infarction |
| I255 | Ischaemic cardiomyopathy |
| I630 | Cereb infarct dt thrombosis precereb art |
| I631 | Cereb infarct dt embolism precereb art |
| I632 | Cereb infarct dt occlus precereb art NOS |
| I633 | Cereb infarction dt thrombosis cereb art |
| I634 | Cereb infarct dt embolism cerebral art |
| I635 | Cereb infarct dt occlusion cereb art NOS |
| I636 | Cereb infarct dt cntrl ven thromb nonpyo |
| I638 | Other cerebral infarction |
| I639 | Cerebral infarction unspecified |
| I64 | Stroke not spec haemorrhage or infarct |
| I650 | Occlusion & stenosis vertebral artery |
| I651 | Occlusion and stenosis of basilar artery |
| I652 | Occlusion and stenosis of carotid artery |
| I653 | Occlus stenos mult & bil precereb artery |
| I658 | Occlusion & stenosis oth precereb artery |
| I659 | Occlusion & stenosis precereb art NOS |
| I660 | Occlusion stenos middle cerebral artery |
| I661 | Occlusion & stenosis ant cerebral artery |
| I662 | Occlusion & stenosis post cereb artery |
| I663 | Occlusion & stenosis cerebellar arteries |
| I664 | Occlus & stenosis mult & bil cereb art |
| I668 | Occlusion & stenosis other cerebral art |
| I669 | Occlusion & stenosis cerebral artery NOS |
| I693 | Sequelae of cerebral infarction |
| I694 | Sequelae of stroke not haem or infarct |
| I698 | Seq oth/unspec cerebrovascular dis |
| I7021 | Atheroscl artery extrem w intermit claud |
| I7022 | Atheroscl artery extrem w rest pain |
| I7023 | Atherosclerosis artery extremity w ulcer |
| I7024 | Atherosclerosis artery extrem w gangrene |
| I713 | Abdominal aortic aneurysm ruptured |
| I714 | Abdominal aortic aneurysm, without mention of rupture |
| I739 | Peripheral vascular disease unspecified |
| I740 | Embolism & thrombosis abdominal aorta |
| I741 | Embolism & thrombosis oth/unspec aorta |
| I742 | Embolism & thromb arteries upp extrem |
| I743 | Embolism & thromb arteries lower extrem |
| I744 | Embolism & thromb arteries extrem NOS |
| I745 | Embolism and thrombosis of iliac artery |
| I748 | Embolism & thrombosis other arteries |
| I749 | Embolism & thrombosis unspecified artery |
| Z951 | Presence of aortocoronary bypass graft |
| Z955 | Presnc coronary angioplasty impl gft |
| Z958 | Presnc oth cardiac vascular impl graft |
| Z959 | Presnc cardiac vascular impl gft NOS |
| E1052 | Type 1 DM w perph angiopathy w gangr |
| E1452 | Unspec DM w perph angiopathy w gangr |
| 3270000 | Carotid bypass using vein |
| 3270001 | Carotid-carotid bypass using vein |
| 3270002 | Carotid-subclavian bypass using vein |
| 3270003 | Carotid-vertebral bypass using vein |
| 3270004 | Aorto-subclavian-carotid bypass usg vein |
| 3270005 | Carotid bypass using synthetic material |
| 3270006 | Carotid-carotid bypass usg synthc matrl |
| 3270007 | Carotid-vertebral byps usg synthc matrl |
| 3270008 | Carotid-subclavian bypass synthc matrl |
| 3270009 | Aorto-carotid byps usg synthc material |
| 3270010 | Aorto-carot-brachial bypass synthc matrl |
| 3270011 | Aorto-subclavn-carot bypass synthc matrl |
| 3270300 | Resection carotid artery w re-anstms |
| 3270800 | Aorto-femoral bypass usg synthc material |
| 3270801 | Aorto-femoro-femoral bypass synthc matrl |
| 3270802 | Aorto-iliac bypass using synthetic matrl |
| 3270803 | Aorto-ilio-femoral byps usg synthc matrl |
| 3271200 | Ilio-femoral bypass using vein |
| 3271201 | Iliofemoral bypass usg synthc material |
| 3271500 | Subclavian-femoral byps usg synthc matrl |
| 3271501 | Subclavian-bifemoral bypass synthc matrl |
| 3271502 | Axillo-femoral bypass usg synthc matrl |
| 3271503 | Axillo-bifemoral byps usg synthc matrl |
| 3271800 | Ilio-femoral crossover bypass |
| 3271801 | Femoro-femoral crossover bypass |
| 3273000 | Mesenteric bypass usg vein single vessel |
| 3273001 | Mesenteric byps synthc matrl, sgl vesl |
| 3273300 | Mesenteric bypass usg vein mult vessels |
| 3273301 | Mesenteric byps synthc matrl, mult vesl |
| 3273600 | Other proc on inferior mesenteric artery |
| 3273900 | Femoral art bypass usg vein above knee |
| 3274200 | Femoral art bypass usg vein below knee |
| 3274500 | Femor art byps ven, tibl/& peroneal art |
| 3274800 | Femoral art byps usg ven w in 5cm ankle |
| 3275100 | Fermoral art byps synthc matrl abv knee |
| 3275101 | Fermoral art byps synthc matrl blw knee |
| 3275102 | Femor art synthc byps tibl &/peronl art |
| 3275103 | Femor art synthc byps w in 5cm ankle |
| 3275400 | Femoro-femoral bypass usg composite gft |
| 3275401 | Femoro-popliteal byps usg composite gft |
| 3275402 | Femor to tibl/peronl art byps compst gft |
| 3275700 | Femoral artery sequential byps usg vein |
| 3275701 | Femoral art sequential byps synthc matrl |
| 3276300 | Other arterial bypass using vein |
| 3276301 | Other arterial byps gft usg synthc matrl |
| 3276302 | Subclavianvertebral bypass using vein |
| 3276303 | Subclavianaxillary bypass using vein |
| 3276305 | Aortocoeliac bypass using vein |
| 3276306 | Aortofemoropopliteal bypass using vein |
| 3276307 | Ilioiliac bypass using vein |
| 3276308 | Poplitealtibial bypass using vein |
| 3276309 | Aortosubclavian bypass usg synthc matrl |
| 3276310 | Subclaviansubclavian byps, synthc matrl |
| 3276311 | Subclavianvertebral bypass synthc matrl |
| 3276312 | Subclavianaxillary bypass, synthc matrl |
| 3276313 | Axilloaxillary bypass usg synthc matrl |
| 3276314 | Axillobrachial bypass usg synthc matrl |
| 3276316 | Aortocoeliac bypass usg synthetic matrl |
| 3276317 | Aortofemoropopliteal byps synthc matrl |
| 3276318 | Ilioiliac bypass using synthetic matrl |
| 3276319 | Poplitealtibl byps usg synthc material |
| 3305000 | Replace popliteal aneurysm using vein |
| 3305500 | Replace popliteal anrysm usg synthc gft |
| 3307500 | Repair of aneurysm in neck |
| 3308000 | Repair of intra-abdominal aneurysm |
| 3310000 | Replace carotid artery aneurysm w graft |
| 3311200 | Replace suprarenal AAA with graft |
| 3311500 | Replace infrarenal AAA with tube graft |
| 3311800 | Replace infrarnl AAA bifur gft iliac art |
| 3312100 | Replace infrarnl AAA bifur gft femor art |
| 3312400 | Replace iliac art aneurysm w graft, unil |
| 3312700 | Replace iliac art aneurysm w graft, bil |
| 3313000 | Exc & rep visc art aneurysm, dir anstms |
| 3315100 | Replace ruptured suprarenal AAA w graft |
| 3315400 | Replace ruptd infrarenal AAA w tube gft |
| 3315700 | Replace ruptd infrarnl AAA w iliac graft |
| 3316000 | Replace ruptd infrarnl AAA w femor graft |
| 3316300 | Replace ruptd iliac art aneurysm w graft |
| 3317800 | Repair of ruptured aneurysm in neck |
| 3318100 | Repair ruptured intra-abdominal aneurysm |
| 3350000 | Carotid endarterectomy |
| 3350600 | Innominate endarterectomy |
| 3350601 | Subclavian endarterectomy |
| 3350900 | Aorta endarterectomy |
| 3351200 | Aorto-iliac endarterectomy |
| 3351500 | Aorto-femoral endarterectomy |
| 3351501 | Ilio-femoral endarterectomy, bilateral |
| 3351800 | Iliac endarterectomy |
| 3352100 | Ilio-femoral endarterectomy, unilateral |
| 3352400 | Renal endarterectomy, unilateral |
| 3352700 | Renal endarterectomy, bilateral |
| 3353000 | Coeliac endarterectomy |
| 3353001 | Superior mesenteric endarterectomy |
| 3353300 | Coeliac & supr mesenteric endarterectomy |
| 3353600 | Inferior mesenteric endarterectomy |
| 3353900 | Endarterectomy of extremities |
| 3354200 | Extended endarterectomy deep femoral art |
| 3354800 | Patch graft of artery using vein |
| 3354801 | Patch graft art usg synthetic material |
| 3354802 | Patch graft of vein using vein |
| 3354803 | Patch graft vein usg synthetic material |
| 3355100 | Procurement vein fm limb f patch graft |
| 3355400 | Endarterectomy w art byps prep f anstms |
| 3530306 | Perc transluminal balloon angioplasty |
| 3530307 | Open transluminal balloon angioplasty |
| 3530400 | PTCA, 1 coronary artery |
| 3530401 | Open TBA of 1 coronary artery |
| 3530500 | PTCA, multiple coronary arteries |
| 3530501 | Open TBA mult coronary arteries |
| 3530906 | PTA perc w stenting, single stent |
| 3530907 | PTA perc w stenting, multiple stents |
| 3530908 | Open TBA w stenting, single stent |
| 3530909 | Open TBA w stenting, multiple stents |
| 3531000 | Perc ins trnslml stent, sgl coron artery |
| 3531001 | Perc ins mult trnslml stnt sgl coron art |
| 3531002 | Perc ins >=2 trnslml stnt coron arteries |
| 3531003 | Open ins trnslml stent single coron art |
| 3531004 | Opn ins mult trnslml stnt sgl coron art |
| 3531005 | Opn ins mult trnslml stnt coron arteries |
| 3531200 | Perc peripheral artery atherectomy |
| 3531201 | Open peripheral artery atherectomy |
| 3531500 | Perc peripheral laser angioplasty |
| 3531501 | Open peripheral laser angioplasty |
| 3845619 | Oth intrathor proc arteries heart wo CPB |
| 3849700 | Coron art byps using 1 saph vein graft |
| 3849701 | Coron art byps using 2 saph vein grafts |
| 3849702 | Coron art byps using 3 saph vein grafts |
| 3849703 | Coron art byps usg >=4 saph vein grafts |
| 3849704 | Coron art byps usg 1 other venous graft |
| 3849705 | Coron art byps usg 2 other venous grafts |
| 3849706 | Coron art byps usg 3 other venous grafts |
| 3849707 | Coron art byps usg >=4 oth venous grafts |
| 3850000 | Coronary artery bypass, using 1 LIMA gft |
| 3850001 | Coronary artery bypass, using 1 RIMA gft |
| 3850002 | Coron artery bypass usg 1 radial art gft |
| 3850003 | Coron art byps usg 1 epigastric art gft |
| 3850004 | Coron art byps usg 1 other arterial gft |
| 3850300 | Coronary artery bypass, using 2 LIMA gft |
| 3850301 | Coronary artery bypass, using 2 RIMA gft |
| 3850302 | Coron artery bypass usg 2 radial art gft |
| 3850303 | Coron art byps usg 2 epigastric art gft |
| 3850304 | Coron art byps usg >=2 oth arterial gft |
| 3850500 | Open coronary endarterectomy |
| 3850700 | Left ventricular aneurysmectomy |
| 3850800 | L ventricular aneurysmectomy w ptch gft |
| 3850900 | Repair of ventricular septal rupture |
| 3863700 | Reop recon occluded coronary artery |
| 9020100 | Coron art byps usg 1 other matrl gft NEC |
| 9020101 | Coron art byps usg 2 other matrl gft NEC |
| 9020102 | Coron art byps usg 3 other matrl gft NEC |
| 9020103 | Coron art byps usg >=4 other matrl gft |
| 9022900 | Other endarterectomy |
| 9023000 | Embolectomy/thrombectomy of other artery |
