# Supplementary material for: Developing a synthetic national population to investigate the impact of different cardiovascular disease risk management strategies: A derivation and validation study
Source: PLoS One. 2017 Apr 6;12(4):e0173170. doi: 10.1371/journal.pone.0173170 (PMC5383032; doi:10.1371/journal.pone.0173170)
Supplement: S2 File — Further information on the Monte Carlo process used during the population development. (DOCX) [file pone.0173170.s002.docx]

## Supporting Information 2.

## Monte Carlo process description

Phases 1-2 are developed from census data in the following format, where the DHB, Age, Sex, Prioritised_ethnic_group, NZDep13_quintile and min age are categorical variables. Regular.smoker, Ex.smoker and Never.smoked.regularly are frequency counts. The total number of people in the row can be found by summing Regular.smoker, Ex.smoker and Never.smoked.regularly variables.


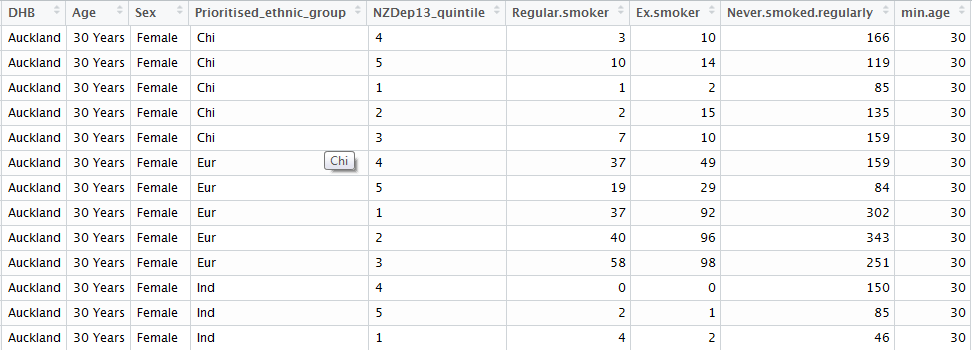


The development process iterates through each row of the census data where the total number of people in the row (as determined by summing the smoking statuses) determines the number of synthetic individuals developed.

For each row of the census data:

1. Assign age by random draw of X values from a uniform distribution bounded by Y and Y+1 where Y is the minimum age group and X is the number of individuals in the age group (as determined by summing the smoking statuses).
2. Sex, prioritised ethnic group and NZDep are then assigned on the basis of the value for the strata in question to all individuals created in step 1. This assignment is deterministic as for each row (see figure above) there is only a single option of both gender and ethnicity.
3. Assign smoking status on the basis of age, sex, ethnicity, NZDep

$$P\left( smoking status | age, sex,ethnicity,\mathrm{NZDep} \right)$$

4. Assign a joint diabetes and CVD status on the basis of age, sex ethnicity and NZDep.

$P\left( Diabetes \cup CVD | age,sex,ethnicity,\mathrm{NZDep} \right)$

1. Assign a joint medication status (LLD = lipid modifying medication, AHT = Antihypertensive medication) on the basis of age, sex ethnicity and CVD status.

$$P\left( LLD\cup AHT | age,sex,ethnicity,CVD \right)$$

2. Multiple imputation is then used to create SBP, TC:HDL ratio and familial history of CVD on the basis of the values developed in steps 1-4. To allow generation of these values the synthetic data was merged with the PREDICT data to provide the SBP, TC:HDL ratio and familial history data to allow the imputation described in appendix 3.

‘Smoking status’

The smoking status of individuals has been developed on the basis of the variables collected during the 2013 census. For use in may CVD risk equations a binary classification of smoking status is required. At this stage the three level (non-smoker, ex-smoker, current smoker) coded 0,1,2 respectively has been retained. This structure maintains the maximum amount of information, in situations where a binary variable is required the current convention is to collapse to a ‘smoking’ variable, consisting of the current smokers and a ‘non-smoking variable’ in consisting of the ex- and non-smokers. This classification however is up to the discretion of the user of the synthetic population based on the use case in question.

‘Not elsewhere included’ variables

Census data for the age and gender variables was complete however individuals without a valid answer in the census for the ethnicity and SES variables were assigned to a ‘not elsewhere included’ category. To allow CVD risk calculation all individuals in the synthetic population needed to be assigned an ethnicity and NZDep status. To integrate the individuals without a valid ethnicity the number of individuals in the ‘not elsewhere included’ ethnicity was assigned to ethnicity categories in the synthetic population based on the proportion of known ethnicities in the census population as stratified by age and sex. Likewise, the number of individuals in the ‘not elsewhere included’ category for NZDep was assigned to a NZDep quintile on the basis on the proportions in the known quintiles. Using this method, the total number of individuals in the population was maintained however the total number recorded in the census sub-populations no longer match the synthetic population due to the addition of the proportional ‘not elsewhere included’ population.

Confidentiality rules in relation to Census data

The census data was provided on the basis of existing rules around the protection of the privacy of individuals. The details of which are provided on the Statistics New Zealand website (<http://www.stats.govt.nz/Census/2013-census/methodology/confidentiality-how-applied.aspx>) two rules in particular applied to the census extract, rules 3 and 4.

Rule 3 states that if a count for a cell is less than 6 it will be replaced with a ‘..C’ value.

Rule 4 states that a random rounding rule will be applied such that values not in base 3 (ie ending in 3,6, or 9) will be rounded to the nearest base 2/3 of the time and to the second nearest base 3 1/3 of the time.

As a value was required for all cells the ..C had to be replaced with a valid value. The data was hierarchical and contained subtotals for individuals levels data. this was used to create an internally consistent system such that the values in the strata would sum to the total of the next strata up. For example a total number of 30 year old females of all ethnicities nationally was provided (called upper strata in remainder of description) and in separate rows data for 30 year old females of all individual ethnicities (called lower strata in remainder of description). Note: the process previously described to manage ‘not elsewhere included’ was undertaken before this step so additional ‘not elsewhere included’ individuals were already distributed at both the upper and lower strata levels.

Step 1. If there are no missing values, skip process and move onto next upper/lower strata pairing.

Step 2. Subtract upper strata value from sum of known lower strata values. In some rare cases, due to the rounding , this was not possible as the calculated value was negative, ie the subtotal of the known values in the sub-strata was greater than the total in the strata above. In these cases the value was set to 0.

Step 3. If a single value from the lower strata is the value from step 2 is assigned to the missing value, process ends. If 2 or more values are missing move onto step 4.

Step 4. Draw random values from a 0-1 uniform distribution and sum the values. Assign the value from step 2 proportionally to the missing values, following by rounding to provide whole ‘individuals’.

Following the process above, which was conducted at the outset of the development, only the lowest strata was used. If an aggregated value(e.g. all 30 year old females) is was recreated by summing the lowest level data to ensure internal consistency.
