# Supplementary material for: Developing a synthetic national population to investigate the impact of different cardiovascular disease risk management strategies: A derivation and validation study
Source: PLoS One. 2017 Apr 6;12(4):e0173170. doi: 10.1371/journal.pone.0173170 (PMC5383032; doi:10.1371/journal.pone.0173170)
Supplement: S3 File — Further information on the imputation process used during the population development. (DOCX) [file pone.0173170.s003.docx]

## Supporting Information 3.

## Imputation process description

The synthetic population was divided into sub-populations consisting of ten-year age groups between 30 and 80 and a final group up to 85. The data was again divided on the basis of age to create 12 separate populations to be imputed. The PREDICT data was similarly divided into 12 subpopulations, to prevent any edge conditions and ensure trends where accurately imputed, the age range was extended 10 years below the bottom of the age range used for the synthetic population and 20 years above the maximum.

The individual sub-populations were then imputed, a single complete imputation extracted and then merged to form a dataset that contained all individuals. Finally, the PREDICT portion of the appended data was removed leaving only the synthetic data. In this way the synthetic population was consistent with PREDICT data as well as preserving the relationships between the existing (age, sex, ethnicity, diabetes and smoking) variables. Multiple imputation has been used before to generate synthetic populations using individual level (also known as micro-level) data [[1](#_ENREF_1)] as well as to analyse CVD data in which there were missing variables [[2](#_ENREF_2)].

The R package MICE [[3](#_ENREF_3)] was used for the multiple imputation. It is based on the idea of chaining variables and developing realistic values for data that is missing. The basis of multiple imputation has been explored in detail elsewhere [[4](#_ENREF_4), [5](#_ENREF_5)] however it is briefly described here. Under a fully conditional specification (FCS) method of multiple imputation (of which MICE is one) the relationship between variable is pre-specified and from this each variable has an imputation model developed. In this case it was on the basis of a minimum Pearson correlation of 0.1 ensuring that variables that have no relationship to the variable of interest aren’t used in the imputation model.

The family of imputation models used in each instance is largely determined by the type of data being imputed; within this population a logistic regression method for the binary variables and a Bayesian linear regression was used for continuous data. A predictive mean matching method would have been used for the continuous variables however was found to be too computationally intensive for the volume of data being processed. An advantage of the predictive mean matching method is that it cannot provide a value that is not found in the original dataset, this is not the case for Bayesian linear regression method. To prevent biologically impossible outliers, values for the continuous data points were constrained to between the minimum and maximum values found in the VIEW dataset. These were 60 – 266 mmHg for systolic blood pressure and 1.058 – 30.1 for TC:HDL ratio.

The process of multiple imputation starts with a value being imputed for each of the missing datum points. Following the process, a second iteration is undertaken where each datum point that was originally missing is iteratively re-imputed on the basis of the current data; which is made up of the data that was originally not missing and the data imputed in the first iteration. This cycle is repeated a number of times to ensure that the imputed data is consistent with both the original data and the newly imputed data. Within this population the number of iterations was set to 15 as this was recommended as a sufficient number of cycles to achieve convergence [[3](#_ENREF_3)]. To improve the quality of the imported variables, other related variables where also imputed for example diastolic blood pressure was imputed to improve the quality of the systolic blood pressure however was not reported on as it is not used in multivariable CVD risk prediction algorithms.

1. Raghunathan TE, Reiter JP, Rubin DB. Multiple imputation for statistical disclosure limitation. Journal of Official Statistics. 2003;19(1):1-16.

2. Van Buuren S, Boshuizen HC, Knook DL. Multiple imputation of missing blood pressure covariates in survival analysis. Statistics in Medicine. 1999;18(6):681-94.

3. Buuren S, Groothuis-Oudshoorn K. MICE: Multivariate imputation by chained equations in R. Journal of Statistical Software. 2011;45(3).

4. Azur MJ, Stuart EA, Frangakis C, Leaf PJ. Multiple imputation by chained equations: what is it and how does it work? International Journal of Methods in Psychiatric Research. 2011;20(1):40-9. doi: 10.1002/mpr.329.

5. Sterne JAC, White IR, Carlin JB, Spratt M, Royston P, Kenward MG, et al. Multiple imputation for missing data in epidemiological and clinical research: potential and pitfalls. British Medical Journal. 2009;338.
