# Supplementary material for: Developing a synthetic national population to investigate the impact of different cardiovascular disease risk management strategies: A derivation and validation study
Source: PLoS One. 2017 Apr 6;12(4):e0173170. doi: 10.1371/journal.pone.0173170 (PMC5383032; doi:10.1371/journal.pone.0173170)
Supplement: S4 File — Description of the adjustments undertake to modify synthetic blood pressure and lipid data. (DOCX) [file pone.0173170.s004.docx]

## Supporting Information 4.

## Blood pressure and TC:HDL ratio adjustment description

**Blood pressure adjustment method:**

*For males:*

Step 1:

Abs(70 – age) * 0.11 + existing systolic BP

Step 2:

If >=40 (age -40) * 0.18 + existing systolic BP

Step 3:

If >=70 existing systolic BP - (age - 70) * 0.4

*For females:*

Step 1:

Existing systolic BP - 3

Step 2:

If >=40 (age -40) * 0.1 + existing systolic BP

Step 3:

If >=70 existing (age - 70) * 0.2 + systolic BP

**TC:HDL ratio adjustment method**

*For males:*

Step 1:

Existing TC:HDL – 0.08

Step 2:

If age<=35, then;

Existing TC:HDL + (Existing TC:HDL * (35 – age) * 0.01)

*For females:*

Step 1:

If age<=45, then;

Existing TC:HDL - (Existing TC:HDL * (45 – age) * 0.04)

Step 2:

If age>=45, then;

Existing TC:HDL + (Existing TC:HDL * (age - 45) * 0.008)
