# Supplementary material for: Developing a synthetic national population to investigate the impact of different cardiovascular disease risk management strategies: A derivation and validation study
Source: PLoS One. 2017 Apr 6;12(4):e0173170. doi: 10.1371/journal.pone.0173170 (PMC5383032; doi:10.1371/journal.pone.0173170)
Supplement: S5 File — Nine additional plots of internal validation exercises undertaken. (DOCX) [file pone.0173170.s005.docx]

## Supporting Information 5.

## Additional internal validation plots excluded from the main text.


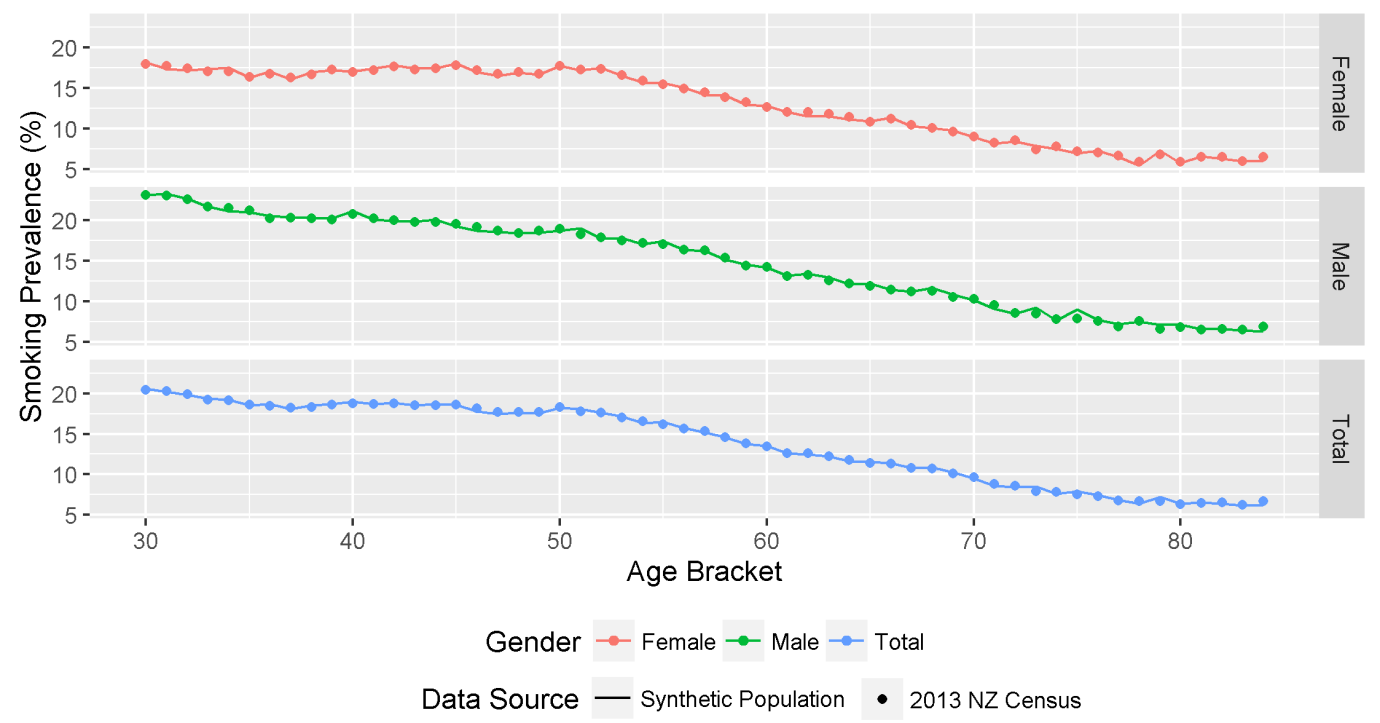
Fig. A Smoking percentage by age and gender

**
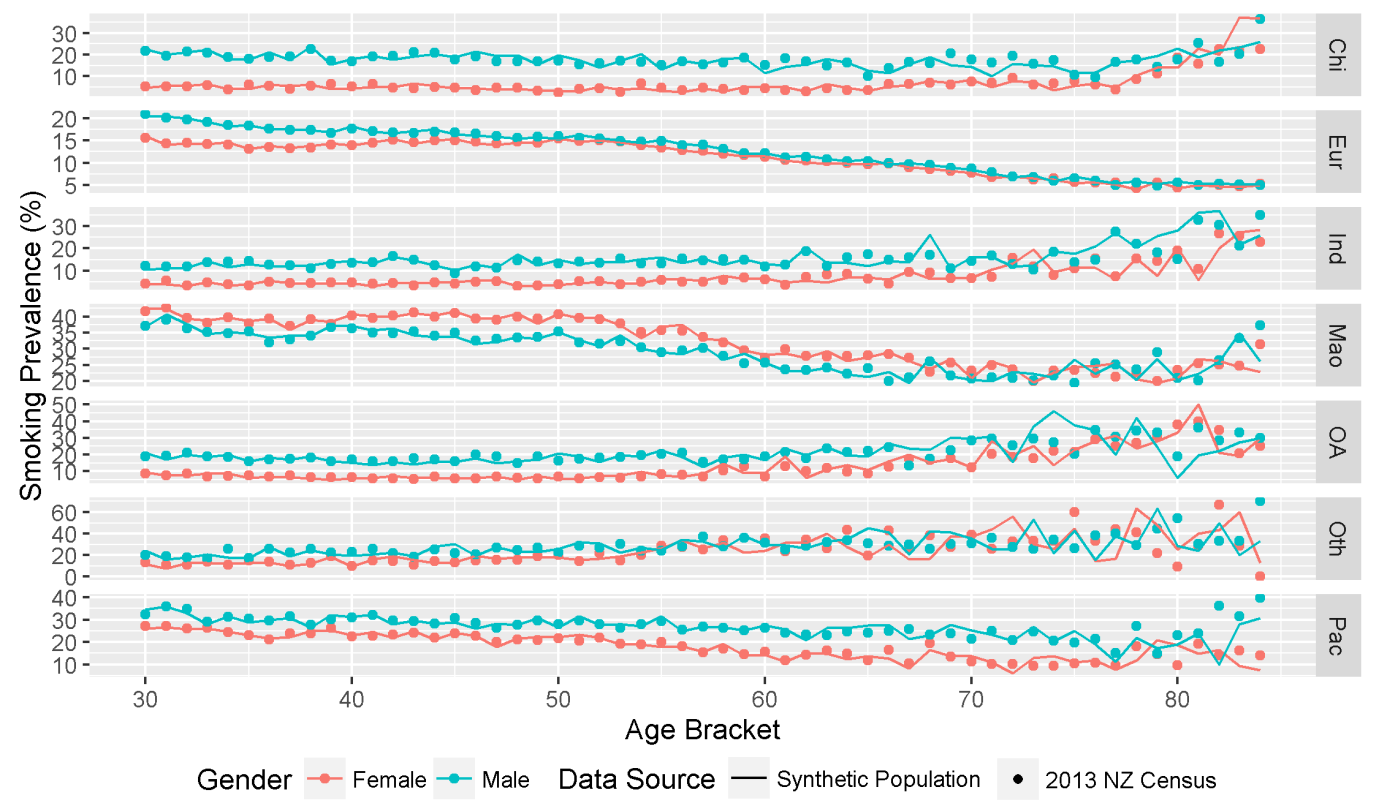
**

Fig. B smoking percentage by age, gender and ethnicity


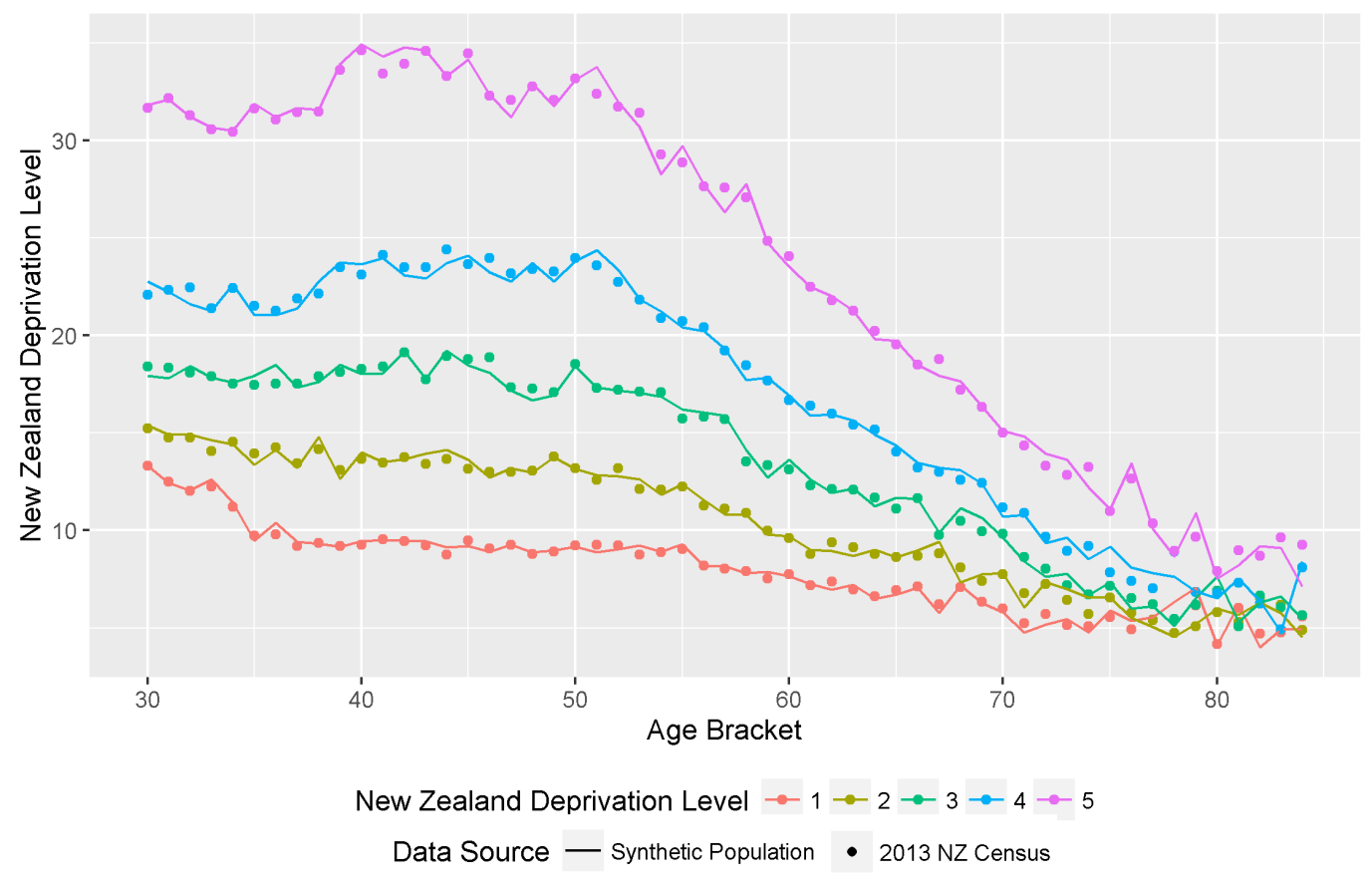
Fig. C smoking percentage by age, and New Zealand deprivation level


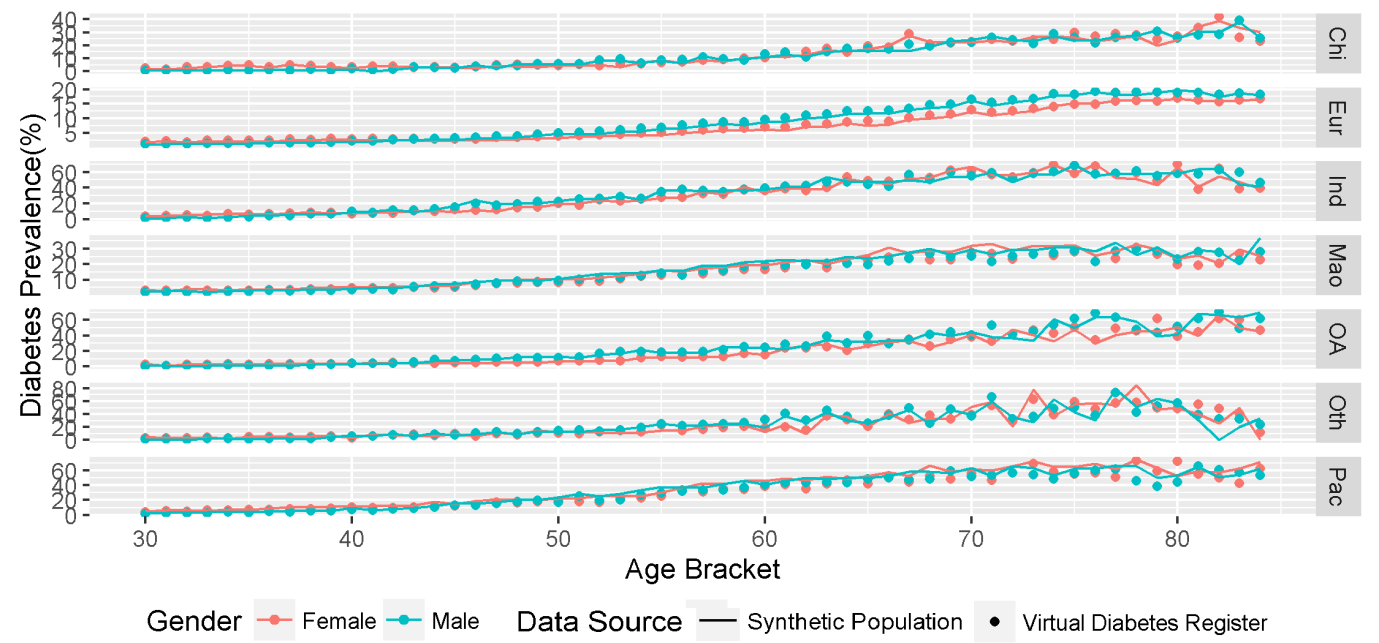


Fig. D Diabetes status by age, gender and ethnicity


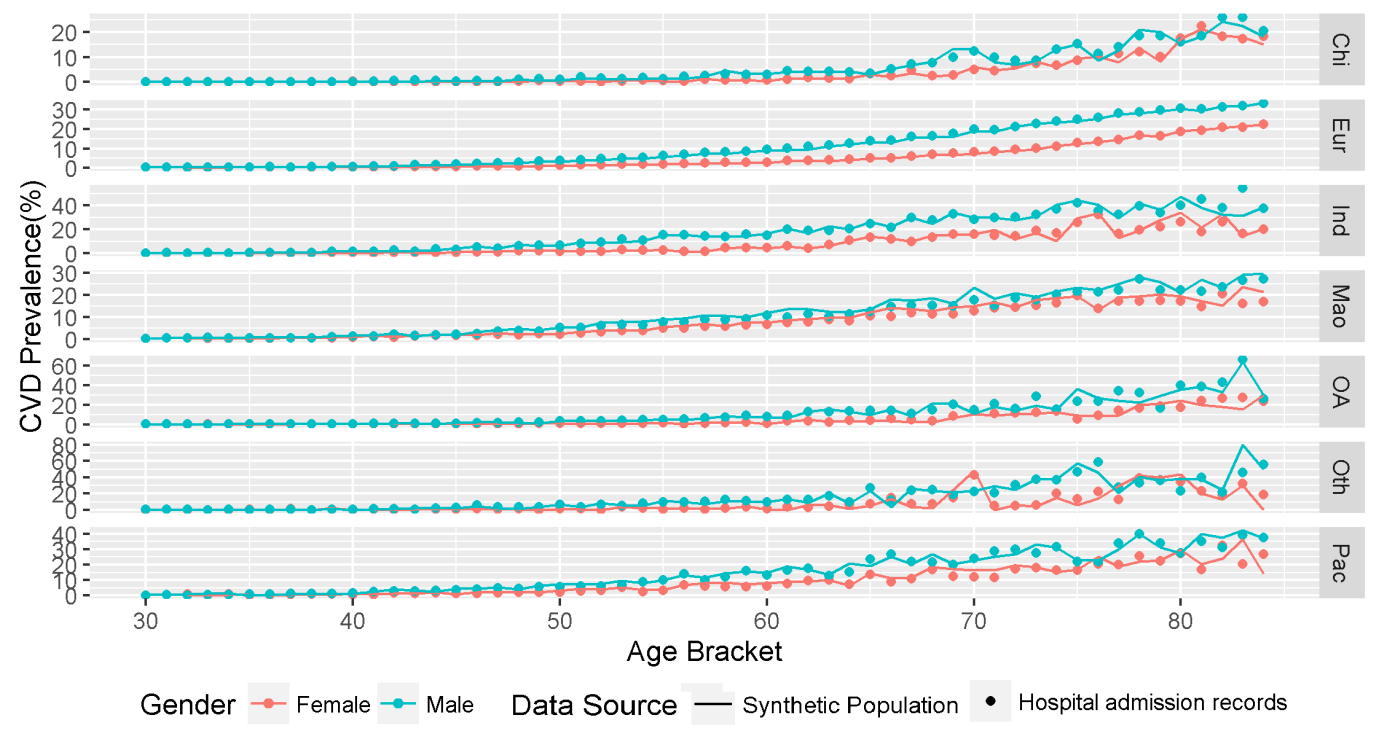


Fig. E History of CVD status by age and ethnicity


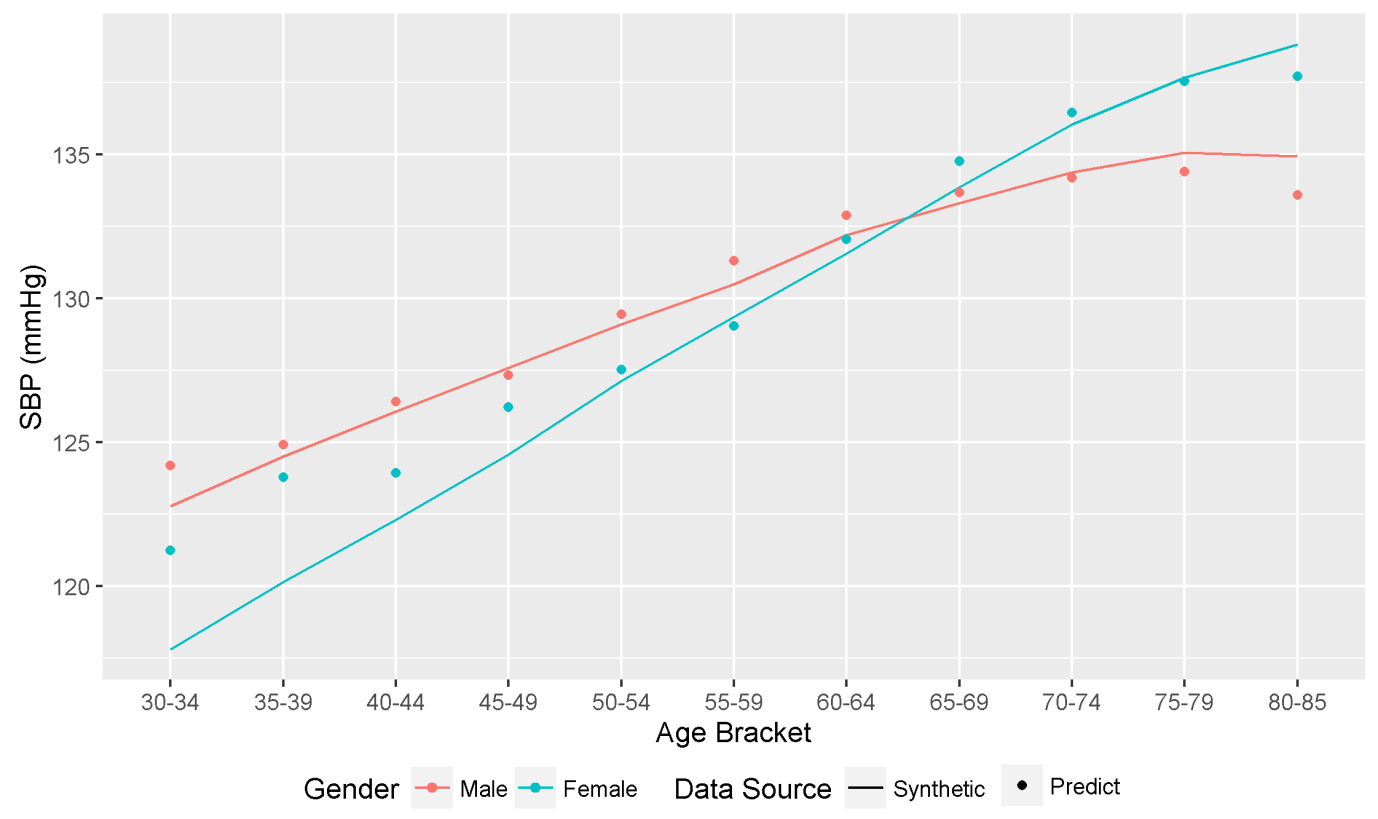


Fig. F Systolic blood pressure by age and gender (pre modification).


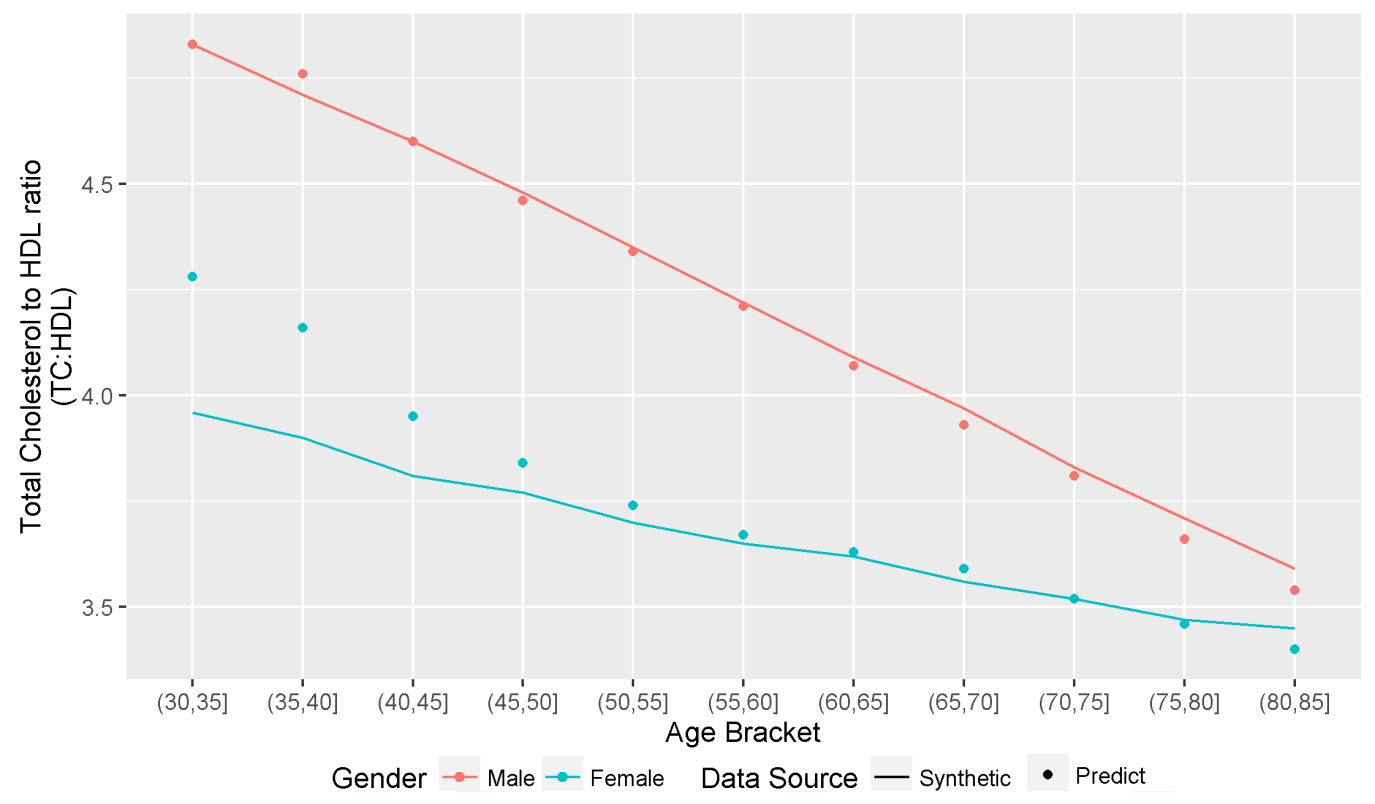


Fig. G Total cholesterol to HDL ratio by age and gender (pre modification).

**
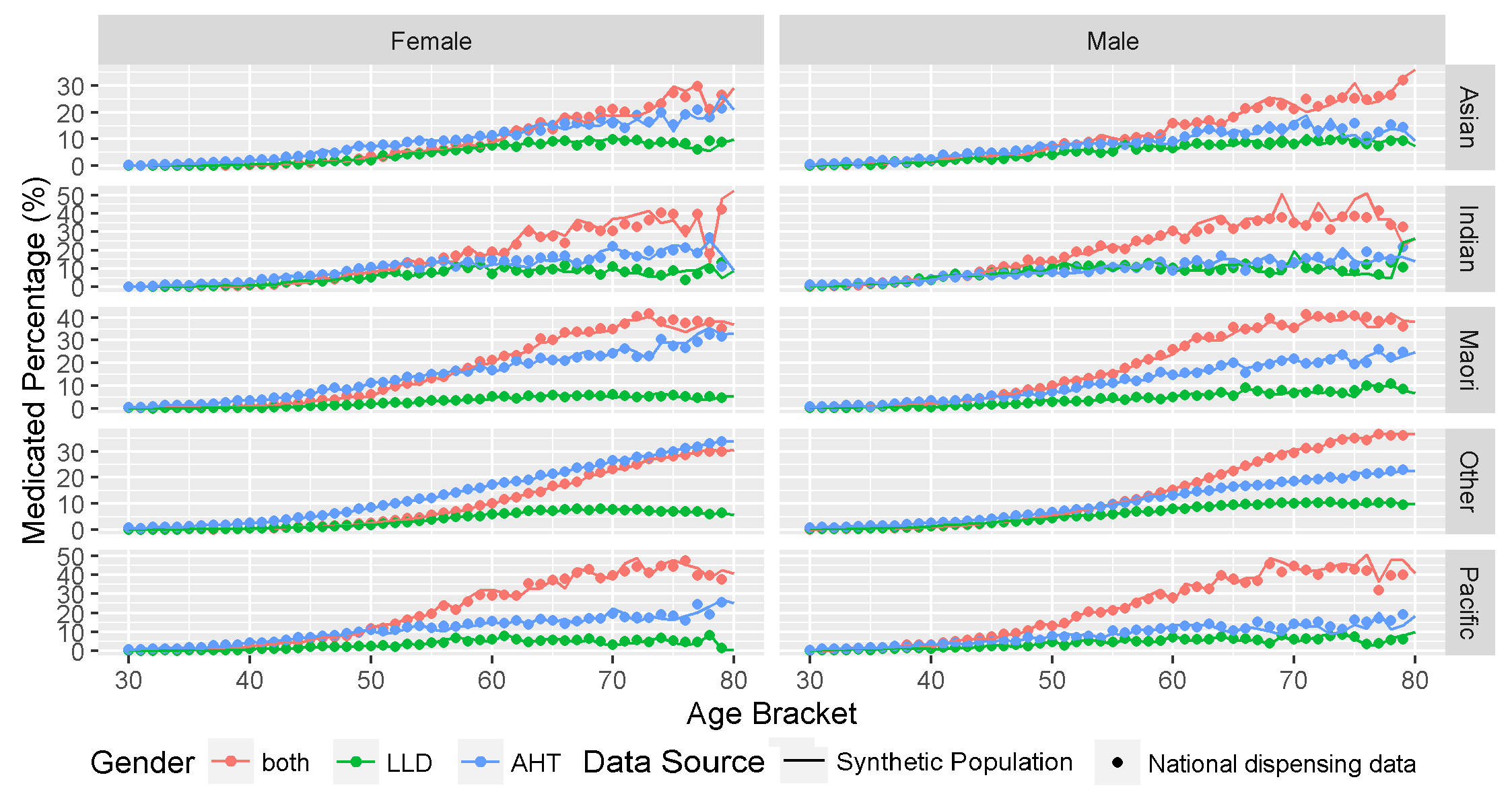
**Fig. H Medication status by age and ethnicity where ‘European’ is included in ‘Other’ ethnicity


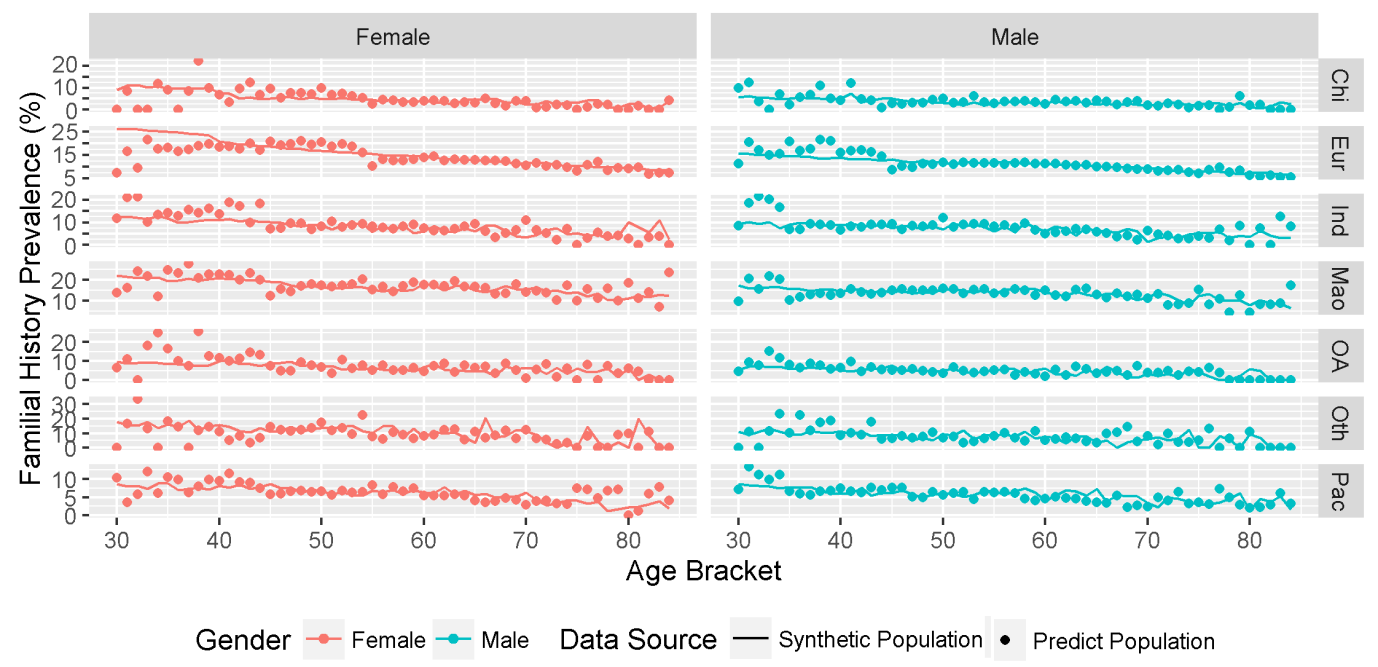


Fig. I Familial history of premature cardiovascular disease
